# Impact of the Synbiotics and Urate-Lowering Therapy on Gut Microbiota and Cytokine Profile in Patients with Chronic Gouty Arthritis

**DOI:** 10.25122/jml-2020-0065

**Published:** 2020

**Authors:** Vitalii Evgenovich Kondratiuk, Oksana Mykhailivna Tarasenko, Olena Myroslavivna Karmazina, Valentyn Valentinovich Taranchuk

**Affiliations:** 1. Department of Internal Medicine No 2., Bogomolets National Medical University; 2. Rheumatology Department, Kyiv City Hospital No 3., Kyiv, Ukraine; 3. Department of Internal Medicine No.1, Bogomolets National Medical University

**Keywords:** Gout, cytokines, fecal microbiota, therapy, synbiotic., BOS - bacterial overgrowth syndrome, CFU - colony-forming unit, HU-hyperuricemia, IL - interleukin, NSAIDs - non-steroidal anti-inflammatory drugs, MSU - monosodium urate, TNF - tumor necrosis factor, UA - uric acid, GIT - gastrointestinal tract, ULT - urate-lowering therapy

## Abstract

The main goal of our study is the impact evaluation of complex urate-lowering therapy with the synbiotic addition on fecal microbiota and cytokine profile in patients with primary gout. During our study, 130 men (mean age 55.5 ± 9.4 years) with gout (duration 7.7 ± 7.1 years) were examined. All patients were divided into two treatment groups. The main group (n = 68) was taking allopurinol at 300 mg per day dose and additionally a synbiotic. The comparison group (n = 62) received allopurinol monotherapy without synbiotic intake. The therapy duration was 3 months. Evaluation of therapy efficiency was marked by blood uric acid changes, cytokine levels, CRP and fecal microbiota condition. After treatment, stabilization of the gut microbiota parameters was observed, which was leading to normalization uricemia levels (40.3% vs. 21%, p <0.01) in the main group patients. Addition of synbiotic to allopurinol leads to a blood uric acid lowering (18.7% vs. 13.3%, p <0.01), CRP reduction (75% vs. 26.3%, p <0.01) as well as decrease of cytokines level: IL-1β, IL-6, IL-8, IL-10 and TNFα (all p <0.001). After a 3-month gout treatment, a group of patients who received complex therapy with synbiotic inclusion showed signs of disease remission characterized by inflammation activity reducing, fecal microbiota condition normalization and a more pronounced decrease in laboratory markers comparing to control group.

## Introduction

Gout is an inflammasome-activated autoinflammatory syndrome, combined with metabolic disorders with urate accumulation due to hyperuricemia (HU). Under appropriate conditions, the latter can appear by articular and extra-articular crystallization with chronic inflammation development [1]. Currently, gout is characterized by a more aggressive course, manifested by involvement in the pathological process of more joints, nephrolithiasis presence, a frequent transition to chronic arthritis, an increased prevalence in the case of women, an expanded frequency of family gout, and growth of costs for treating patients [2, 3]. Up to 4% of the world population have gout, and 4-20% have HU [1]. In 10% of people with HU, gout is in the forming stage; 80-90% of patients with gout have HU [4, 5]. The uric acid (UA) concentration in the serum is age- and sex-dependent. UA reference values in men’s blood are higher (up to 7 mg/dL) than in women of childbearing age (up to 6 mg/dL). HU is a level of uricemia above 6.8 mg/dL when the in vitro solubility of monosodium urate (MSU) is limited [6, 7].

The exchange of urates depends on the balance between the summability of the renal tubules secretion, excretion processes, and the gastrointestinal tract (GIT) activity. Urates excretion is performed in two ways: by kidneys (65-75%) and GIT (25-35%) [8]. The bacterial uricolysis of UA to allantoin and CO2 by bacterial transporters is performed by the GIT [6]. Different bacteria colonizing the colon are using UA as a metabolic substrate. E. coli, lactobacilli, and Pseudomonas contribute dissociation of purines to allantoin, allantoinase, and urea by enzyme xanthine oxidase synthesizing [9]. Intestinal dysbiosis in gout patients is caused by the prevalence of Bacteroides caccae and Bacteroides xylanisolvens compared to bacteria of healthy people. Such a process is associated with increased microbial xanthine dehydrogenase and lower microbial allantoinase level that needs correction [10].

Probiotics represent living microorganisms’ strains, mainly lactic- and bifidobacteria, which show anti-inflammatory and hypouricemic qualities in mice experimental studies [11-17]. Some researchers believe that probiotics make a linkage with some of the compounds required for UA synthesis. Other probiotic strains (especially those that contain lactobacilli) contribute to the intermediate purine’s forms degradation (inositol and guanosine) [18-20]. By reducing the intake of purine nucleotides, the decrease in AU synthesis is expected. During an experimental study on rats by using genetic engineering technology, a probiotic that contained the urease gene with the inclusion of DH5 Escherichia coli was created, leading to uricemia level lowering [9, 21]. E. coli can use allantoin as a source of nitrogen under anaerobic conditions. Genes encoding allantoin and glyoxylic acid metabolism enzymes are linked and controlled by the product of the allR gene. Allantoin and glyoxylic acid are used as effector molecules [22, 23].

In another work, a capsule form of a probiotic containing an L. fermentum ATCC 11976 strain was used, showing a decaying property [2]. Another study showed a reduction of inflammatory activity after patients received a probiotic containing a Bifidobacterium longum 51A strain [23].

Prebiotics are non-digestible dietary fibers that are selectively stimulating the definite bacterial groups’ growth and activity with beneficial metabolites synthesis [24]. Prebiotic components improve immune system functioning by affecting the cytokine profile [25-27]. Inulin is a prebiotic which has the unique property of inhibiting xanthine oxidase activity, reducing the level of UA in the blood. Moreover, inulin reduces total cholesterol and can improve carbohydrate metabolism [28]. Useful prebiotic properties are conditioned by intestinal barrier function optimization and improvement of the immune system functioning, a decrease of Clostridium species, which are pathogenic subpopulations, increase of symbiotic flora growth (Lacto- and bifidobacteria), enhancing short-chain fatty acids production [29].

Current gout therapy is based on permanent basic urate-lowering therapy (ULT) usage by xanthine oxidase inhibitors (allopurinol, febuxostat) in order to achieve the target level of uricemia. However, their effectiveness is completed only by activating the renal excretion of UA. The extrarenal pathway of UA excretion in gout patients is not used during urate-lowering therapy. Nevertheless, bacterial uricolysis is working [10, 30], which makes an urgent question of finding extrarenal UA excretion ways through the correction of the gut microbiota. One way of solving this problem is the engagement of synbiotics in gout treatment. Considering the positive impact of synbiotics on cytokine profile, immune system optimization, and uricemia level [19, 25, 26], scientific search in this direction seems promising to improve the results of patients receiving gout treatment.

## Material and Methods

### Inclusion criteria

The given study presents the results of 130 men with gout, receiving treatment and post-hospital observation at the No. 3 Rheumatology Department of Kyiv City Hospital. Eligibility criteria were the following: age: 18 - 75 years old; gout diagnostic according to the American College of Rheumatology (ACR) criteria from 2016; disease course in the remission phase; consciousness and ability to provide written informed consent; the ability to perform research requirements. Exclusion Criteria were: diseases leading to secondary HU and cause elevated blood interleukins such as myeloproliferative diseases, hemolytic anemia, psoriasis, sarcoidosis, acute and chronic renal failure, type 1 and 2 diabetes mellitus, hypo- and hyperthyroidism, GIT cancer, peptic ulcer, bacterial overgrowth syndrome (BOS), inflammatory bowel diseases (nonspecific ulcerative colitis and Crohn’s disease), opportunistic infections, intake of any drugs other than allopurinol urate-lowering agents, glucocorticoids, treatment with non-steroidal anti-inflammatory drugs (NSAIDs), proton-pump inhibitors, antibiotics, laxatives, other pre- or probiotics; alcohol and/or drug abuse, mental illness, participation in other clinical trials. The clinical study was conducted by the ethical principles of the Declaration of Helsinki. An informed agreement was obtained from all patients before entering the study.

Patients were divided into two groups: main (n = 68) and comparison group (n = 62). The general characteristics of the two groups are presented in Table 1.

**Table 1:** General data of the patients.

Indicator	Group	
	Main (n = 68)	Control (n = 62)
**Average age, years**	55.5 (47.00;61.5)	57.00 (48.00;63.00)
**Gout duration, years**	6.0 (3;8)	6.0 (3;10)
**Tophus gout, abs. (%)**	19 (27.9)	20 (32.2)
**Gout without tophus, abs. (%)**	49 (72.1)	42 (67.8)
**Uric acid, mmol/l**	455.00 (398.50;531.00)	465.5(406.00;546.00)
**BMI, kg/m2**	29.45 (27.45;32.00)	30.55 (27.40;33.80)
**Stage I, abs. (%)**	9 (13.2)	11 (17.7)
**Stage II, abs. (%)**	34 (50)	27 (43.5)
**Stage III, abs. (%)**	23 (33.8)	21 (33.9)
**Stage IV, abs. (%)**	2 (2.9)	3 (4.8)

BMI - body mass index.

Before day 0, all patients underwent 6 weeks of allopurinol therapy without uricemia reference values reaching. Further, patients from the main group continued to receive a daily 300 mg dose of allopurinol, with dose titration up to 100 mg every month and additional synbiotic intake according to the standard scheme. The Rotabiotic synbiotic was used, containing lyophilized bacteria 2.5 × 109 colony forming units (CFU): Lactobacillus bulgaricus - 0.5 × 109 CFU, Streptococcus thermophilus - 0.8 × 109 CFU, Lactobacillus acidophilus - 0.8 × 109 CFU, Bifidobacterium s. (B. bifidum, B. longum, B. infantis) - 0.4 × 109 CFU; inulin - 150.0 mg in one hard capsule. The comparison group of patients continued allopurinol monotherapy, according to a similar scheme after day 0. The observation duration was 3 months. The control group consisted of 25 almost healthy volunteers appropriate by age and gender, without arthritis history. The treatment scheme’s effectiveness was evaluated by comparing the dynamics of clinical and laboratory parameters between the patients of the main and comparison groups during the 3 months of treatment (after day 0 and after month 3). C-reactive protein levels, uricemia, and cytokine profile before and after therapy were the evaluated laboratory parameters. Gut microbiota qualitative and quantitative indicators estimation in gout patients was provided by feces material sowing before and after treatment according to the standard method. Immunological changes in gout patients were evaluated by cytokines concentrations (IL-1β, IL-6, IL-8, IL-10, TNFα). Serum cytokines determination was carried by enzyme-linked immunosorbent assay (ELISA) using Vector-Best reagents (Russia) on a PR2100 reader (Sanofi Diagnostics Pasteur, France).

### Statistical analysis

When evaluating each group of indicators, the type of distribution of the indicator was evaluated, the results being normal or different from normal. Distribution uniformity was estimated by Shapiro-Wilk test exploitation. According to the homogenous distribution of exploring indicators, the following statistical methods were applied: the mean value (M), standard deviation (SD), standard error (SE), 95% mean confidence interval (95% CI). Two independent groups comparison was provided by Student’s t-test. Non-parametric indicators were used: the median (Me), 25 and 75 quartiles (0.5L; 0.5U) for distribution of different from normal indicators. The Mann-Whitney test was used for the two independent groups comparison. Qualitative binary data comparison was performed by Pearson’s χ2 test (Yates correction, in cases where at least one group was less than 10) and Fisher’s exact test. Pearson’s parametric correlation method was used for linear relationships of quantitative, normally distributed data estimation. Spearman correlation analysis was used to evaluate qualitative traits associations, traits with a distribution that is different from normal traits, or traits with the uncertain distribution. The coefficient of rank correlation (r) was calculated to determine the presence and strength of the correlation between factors. Data were considered statistically significant at a value of the statistical significance coefficient p <0.05 and processed using Microsoft Office Excel 2010 and IBM Statistics SPSS v22.

## Results and Discussion

The gut microbiota in gout patients differs from healthy people by an increased number of certain species of Bacteroides and reduced levels of p. Faecalibacterim and Bifidobacterium spp.

Lactobacillus spp. or Pseudomonas spp. produce enzymes capable of converting UA to urea [19, 31]. Intestine dysbiotic changes in gout patients are characterized by a combination of protective microflora deficit (lactobacilli and bifidobacteria reducing level) and increased intestine contamination level with obligate anaerobes, pathogenic Enterobacteriaceae, gram-positive cocci, and different Candida types.

During the 3-month complex treatment of the gout patients from the main group with the synbiotic addition, there was a significant transformation of the intestinal microbiota structure towards normalization of its qualitative and quantitative composition. The tendency of protective microflora restoration is observed through the normal reference values of lactobacilli and normalization of intestinal bifidobacteria contamination level (Tables 2 and 3).

**Table 2:** Dynamics of intestinal facultative anaerobic bacteria spectrum in main group after treatment with the synbiotic addition (lg CFU/g), Me, 0.5 L; 0.5 U (Q1;Q3).

Microorganisms	Main group (n=68)			Control group	
		3 months		(n=25)	
	lg	lg CFU/g	p1	lg CFU/g	p2
E. coli	6 (6;7)	8 (8;8)	0.000	8 (8;8)	0.037
E. coli with altered enzymatic functions	6 (6;7)	3 (3;5)	0.028	4.5 (3;6)	0.846
Non- lactose E. coli	7 (6;7)	3 (3;3)	0.019	3 (3;3)	0.816
E. coli (heme+)	7 (7;7)	-	1.000	-	1.000
Klebsiella spp.	7 (7;7)	3 (3;5)	0.000	3 (3;6)	1.000
Citrobacter spp.	6 (7;7)	3 (3;5)	0.003	3 (3;3)	1.000
Proteus spp.	7 (6;8)	-	1.000	-	1.000
Enterobacter spp.	7 (7;7)	3 (3;3)	0.001	3 (3;3)	0.867
S. aureus	5 (5;5)	3 (3;4)	0.001	3.6(3.2;4)	1.000
S. epidermidis (heme+)	5 (5;5)	3 (3;4)	0.002	3 (3;3)	1.000
S. saprophyticus	3 (3;3)	3 (3;3)	0.589	3 (3;4)	0.311
S. faecalis	5 (5;5)	6 (6;6)	0.000	6 (5;7)	0.314
Different Candida kinds	5 (5;5)	4 (3;5)	0.001	3 (3;3)	0.125
Lactobacillus spp.	5 (5;5)	8 (8;8)	0.000	8 (8;8)	0.376

p1 - statistical reliability between the main group flora parameters before and after therapy; p2 - the statistical significance between the main and control group flora indices.

Lowering of Gram-positive conditionally pathogenic microorganisms such as Firmicutes and Candida kinds (by 17.7%) was indicated in the main group of patients after treatment. Main group representatives reached reference values levels of healthy individuals, except the E. coli CFU/g indicator.

There were no positive changes in the spectrum of obligate anaerobes after synbiotic addition in gout patients (Table 3). A statistically significant decrease in Bacteroides spp. and an increase in the Bifidobacteria number close to control group values were registered.

**Table 3:** Dynamics of intestinal obligate anaerobic bacteria spectrum in main group after treatment with the synbiotic addition (lg CFU/g), Me, 0.5 L; 0,5 U (Q1;Q3).

Microorganisms	Main group (n=68)			Control group	
	day 0	3 months		(n=25)	
	lg CFU/g	lg CFU/g	p1	lg CFU/g	p2
Bacteroides spp.	12 (12;12)	10 (10;11)	0.000	9 (9;9)	0.000
Peptostreptococcus spp.	7 (6;8)	6 (6;9)	0.322	6 (6;6)	0.761
Veilonella spp.	10 (9;12)	7 (6;10)	0.007	9 (8;9)	0.235
Fusobacterium spp.	11 (10;12)	8 (8;9)	0.007	8 (8;8)	0.268
Eubacterium spp.	8 (7;11)	9 (9;12)	0.182	10 (10;11)	0.279
Bifidobacterium spp.	6 (6;6)	9 (8;10)	0.000	11 (9;11)	0.000

p1 - statistical reliability between the main group flora parameters before and after therapy; p2 - the statistical significance between the main and control group flora indices.

The obtained data indicate a positive effect of synbiotic therapy on the gut microbiota condition in gout patients, manifested by reducing the imbalance between representatives of the protective stabilizing microflora, conditionally pathogenic aerobic and obligate anaerobic microflora of the gut.

ULT conducted without synbiotic addition had no impact on the gut microbiota (Tables 4 and 5). Therefore, no significant improvement in fecal microflora was observed in the comparison group after the course of therapy without synbiotic usage. The restoration of the protective microflora level was not observed. The quantitative intestinal contamination indicators with conditionally pathogenic facultative anaerobes and obligate anaerobic microorganisms remained high.

**Table 4:** Dynamics of intestinal facultative anaerobic bacteria spectrum in the comparsion group after treatment with the synbiotic addition (lg CFU/g), Me, 0.5 L; 0.5 U (Q1;Q3).

Microorganisms	Comparison group (n=62)			Control group (n=25)	
	day 0	3 months			
	lg CFU/g	lg CFU/g	p1	lg CFU/g	p2
**E. coli**	6 (6;7)	6 (6;7)	0.925	8 (8;8)	0.000
**Facultative E. coli**	6 (6;6)	6 (3;7)	0.916	4.5 (3;6)	0.698
**Non- lactose E. coli**	6 (3;7)	7 (5;7)	0.595	3 (3;3)	0.247
**E. coli (heme+)**	7 (7;7)	7 (6;7)	0.176	-	1.000
**Klebsiella spp.**	6 (3;7)	3 (6;7)	0.891	3 (3;6)	0.067
**Citrobacter spp.**	7 (7;7)	7 (7;7)	0.715	3 (3;3)	1.000
**Proteus spp.**	6,5 (6;7)	6 (6;6)	0.301	-	1.000
**Enterobacter spp.**	6 (6;7)	6 (6;7)	0.438	3 (3;3)	0.067
**S. aureus**	5 (5;6)	5 (5;5)	0.162	3.6(3.2;4)	0.056
**S. epidermidis (heme+)**	5 (5;5)	5 (5;5)	0.566	3 (3;3)	0.029
**S. saprophyticus**	3 (3;4)	4 (3;4)	0.350	3 (3;4)	0.515
**S. faecalis**	5 (5;6)	6 (5;6)	0.462	6 (5;7)	0.085
**Different Candida kinds**	5 (5;5)	5 (5;5)	0.517	3 (3;3,3)	0.006
**Lactobacillus spp.**	5 (5;6)	5 (5;6)	0.415	8 (8;8)	0.000

**Table 5:** Dynamics of intestinal obligate anaerobic bacteria spectrum in the comparison group after treatment with the synbiotic addition (lg CFU/g), Me, 0.5 L; 0.5 U (Q1;Q3).

Microorganisms	Group				
		Comparison (n=62)		Control (n=25)	
	day 0	3 months.			
	lg CFU/g	lg CFU/g	p1	lg CFU/g	p2
**Bacteroides spp.**	12(12;12)	12(12;12)	0.330	9(9;9)	0.0001
**Peptostreptococcus spp.**	8 (8;12)	8 (8;10)	0.846	6 (6;6)	0.0001
**Veilonella spp.**	10 (10;12)	10 (10;10)	0.254	9 (8;9)	0.027
**Fusobacterium spp.**	10 (8;12)	12(10;12)	0.275	8 (8;8)	0.001
**Eubacterium spp.**	8 (6;8)	8 (8;9)	0.377	10 (10;11)	0.013
**Bifidobacterium spp.**	6 (6;6)	6 (6;6)	0.650	11 (9;11)	0.0001

The next stage of our research concerned the comparative analysis of the chronic inflammation indicators level in the main and control groups. Levels of CRP and uric acid in patients with remissive gouty arthritis were statistically significantly different from similar control group indicators (Table 6). Gouty arthritis inflammation is induced by monosodium urate (MSU) crystals deposition in the joints and extra-articular tissues. Set inflammatory cascade causes proinflammatory cytokines release and neutrophils and macrophages translocation into the affected joint with MSU crystals phagocytosis [14]. Activated macrophages create a framework for the formation of specific inflammasome proteins triggering transformation mechanisms of inactive pro - IL - 1β into biologically active IL - 1β [31, 32]. IL - 1β causes the wide range of inflammatory mediators release -TNF-α, IL-6, IL-8, IL-1β, which are responsible for the translocation of neutrophils, prostaglandins, kinins, toxic oxygen radicals, activation of the Hageman factor and complement system into the synovial environment [22, 33, 34]. Moreover, according to some studies, IL-6 enhances the inflammatory process and strengthens bone destruction, so this is a treatment target [35].

**Table 6:** Comparative characteristics of the cytokine profile at day 0 of the research and control group (Me, 0.5 L; 0.5 U) (Q1;Q3).

Indicator	Research group (n=130)	Control group (n=25)	P-value (between groups)
UA, mmol/l	459 (399;536)	276 (234;283)	0.000001
Blood CRP, mg/l	11.9 (6;24)	1.3 (0.9;1.7)	0.000001
**IL-1**^b^**, pg/ml**	128 (127;129)	23 (21;24)	0.000001
IL-6, pg/ml	214 (212;215)	22 (21;24)	0.000001
IL-8, pg/ml	43 (43;43)	24 (23;24)	0.000001
**TNF-**α**, pg/ml**	121 (121;122)	25 (24;25)	0.000001
IL-10, pg/ml	72 (71;73)	23 (21;23)	0.000001

Our work presents a certain prevalence of high cytokine profile values in gout patients compared to volunteers. During our research, IL-6, unlike other proinflammatory cytokines, turned out as the most informative indicator in patients with primary gout, increasing up to 10 times compared with healthy individuals. It can be assumed that IL-6 is a marker of severity and aggressive course in chronic gouty arthritis patients. Received data emphasizes that even in the remission phase, gout patients need medical correction, considering the pathophysiological cascade features of specific inflammatory changes.

Uricemia levels analysis in both groups showed no difference before treatment.

**Figure 1:**
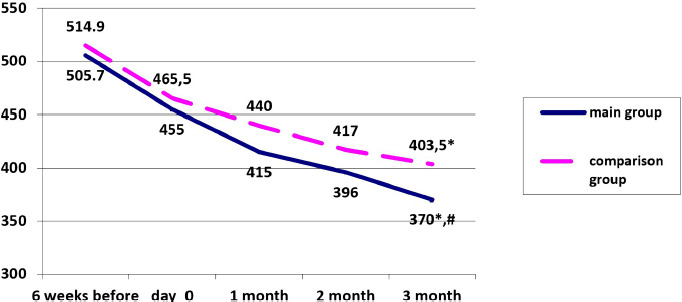
Dynamics of a median level in the main and the comparison group receiving the therapy. * - a significant difference in the levels of uricemia in the main group and the comparison group on day 0 and after 3 months; # - a significant difference in uricemia levels at 3 months between the main and the comparison group.

Uricemia reduction in the main group and the comparison group after 3 months of therapy was registered (18.7% vs. 13.3%, respectively, p <0.01). There was a statistically significant difference in UA levels between the main and comparison groups after 3 months of therapy (p = 0.0014) (Figure 1). In the main group of patients, the level of UA was 360 mmol/l and below - 40.3%. However, in the comparison group, it was only 21.0%.

**Figure 2:**
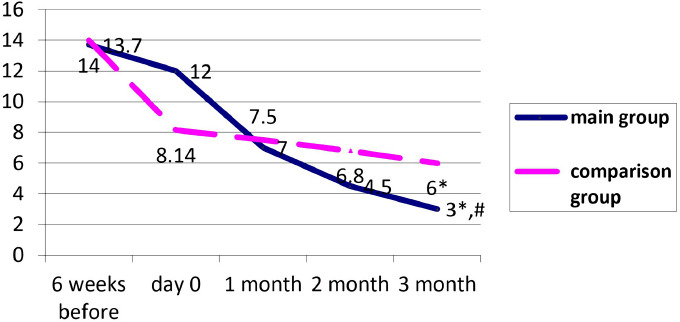
Dynamics of median CRP levels in the main and comparison group receiving the therapy. * - a significant difference in the CRP levels in the main and comparison group on day 0 and after 3 months; # - a significant difference in CRP levels at 3 months between the main and comparison group.

The dynamics of CRP levels in both study groups during treatment are presented in Figure 2.

The CRP level before therapy is comparable in both groups. The 3 months of treatment showed a CRP lowering in both groups; however, more significant dynamics were observed in the main group (75% and 26.3%, respectively, p <0.01). After completing the therapy, the CRP level was significantly lower in the main group than the comparison group (p = 0.016) (Figure 2). CRP lowering rate in both groups was noticeably faster than the uricemia lowering rate. Consequently, it can be assumed that a prerequisite of uricemia target level achievement in gout patients is inflammation activity reduction.

By analyzing the cytokine profile dynamics, the following results were obtained. The concentration of proinflammatory cytokines such as IL-1β, IL-6, IL-8, IL-10, and TNFα in the main group after treatment decreased significantly; The biggest regression was observed in the case of IL-6 - by 17.8% (Table 7). In the comparison group, there was only a tendency of proinflammatory cytokines IL-1 β, TNF-α and IL-6 lowering during interleukin blood values assessment after therapy, explained by the standard urate-lowering monotherapy in this group of patients (Table 7). By analyzing the statistical difference between the interleukin’s indices of both groups after therapy, it is highly reliable only between IL-1β and IL-6. However, the difference between IL-8, IL-10, and TNFα did not reach statistical significance. Patients of both experimental groups receiving the treatment did not reach pro- and anti-inflammatory cytokines reference values due to the long-term inflammatory process persistency in chronic gout patients.

**Table 7:** Comparative characteristics of cytokine profile levels in the main and comparison groups on day 0 and month 3. Me, 0.5 L; 0.5 U (Q1;Q3).

Indicator,pg/ml	Main group (n = 68)			Comparison group (n = 62)		
	Day 0	Month 3	p	Day 0	Month 3	p
**IL-1β**	129 (127;129)	126 (111;127)	0.0001	128 (128;129)	128 (127;129)	0.0599
IL-6	214 (212;215)	176 (176;177)	0.0001	214 (213;215)	212 (211;215)	0.0607
IL-8	43 (43;44)	42 (42;43)	0.0004	43 (43;43)	43 (42;43)	0.2343
**TNF-α**	121 (121;122)	121 (111,5;121)	0.0001	121 (121;121)	121 (121;121)	0.0501
IL-10	72.5 (71;73)	71 (71;72)	0.0002	71 (71;73)	71 (71;73)	0.4061

**Figure 3:**
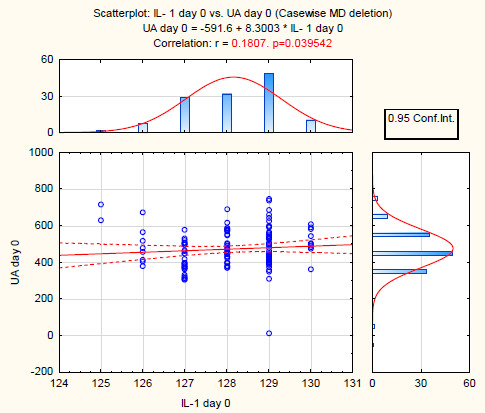
Correlation between uricemia and IL-1β in the study group.

The correlation relationships showed considerable data after the estimation of the results. A weak direct correlation between IL-1β and uricemia in the total cohort (n = 130) was found, indicating an inflammatory growth activity with increased uric acid levels in the blood (Figure 3).

**Figure 4:**
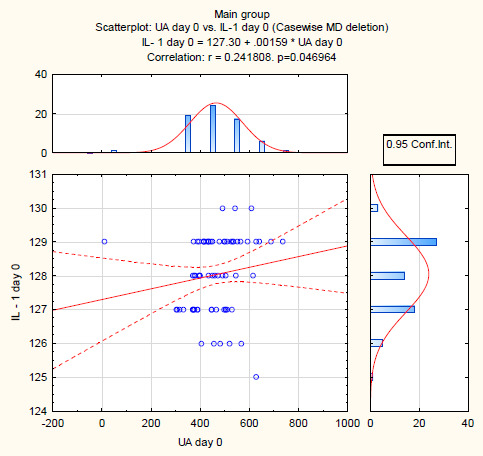
Correlation between uricemia and IL-1β in the main group of patients (n = 68).

In the main group of patients, a more correlated relationship between uricemia and IL-1β was also observed (Figure 4).

Consequently, patients with primary chronic gout show a significant increase in the concentration of IL-1β, IL-6, IL-8, IL-10, and TNFα, even in the remission phase. IL-6 should be considered as the most informative indicator for the evaluation of activity, disease aggressiveness, and therapy effectiveness of chronic gouty arthritis patients.

## Conclusions

Patients with primary gout, beyond the main disease features, also suffer from gut microbiota impairment characterized by a combination of protective microflora deficiency, increased levels of obligate anaerobes, Gram-positive cocci, different Candida kinds. As a result, the proinflammatory cytokine concentration is increased. In patients with gouty arthritis, after three months of complex therapy that included synbiotics, along with normalization of the qualitative and quantitative composition of fecal microbiota, a more pronounced urate-lowering effect was found - uricemia levels were within the normal values two times more often compared with the allopurinol monotherapy. Therefore, normalization of CRP levels and a decrease in the values of proinflammatory cytokines were achieved. IL-6 can be considered a marker of activity, aggressiveness of the disease course, and treatment effectiveness in gout patients. Changes in serum proinflammatory cytokines, gut microbiota after receiving ULT therapy with synbiotics reflect the significant role of the relationship between cytokine status and intestinal dysbiosis in the pathogenesis of primary gout.

## Conflict of Interest

The authors declare that there is no conflict of interest.
